# Altered Regional and Circuit Resting-State Activity Associated with Unilateral Hearing Loss

**DOI:** 10.1371/journal.pone.0096126

**Published:** 2014-05-01

**Authors:** Xingchao Wang, Yang Fan, Fu Zhao, Zhenmin Wang, Jianqiao Ge, Kai Zhang, Zhixian Gao, Jia-Hong Gao, Yihong Yang, Jin Fan, Qihong Zou, Pinan Liu

**Affiliations:** 1 Department of Neurosurgery, Beijing Tiantan Hospital, Capital Medical University, Beijing, China; 2 MRI Research Center and Beijing City Key Lab for Medical Physics and Engineering, Peking University, Beijing, China; 3 Department of Neural reconstruction, Beijing Neurosurgery Institute, Capital Medical University, Beijing, China; 4 McGovern Institute for Brain Research, Peking University, Beijing, China; 5 Neuroimaging Research Branch, National Institute on Drug Abuse, National Institutes of Health, Baltimore, Maryland, United States of America; 6 Department of Psychology, Queens College, The City University of New York, Flushing, New York, United States of America; 7 Department of Psychiatry, Mount Sinai School of Medicine, New York, New York, United States of America; 8 Friedman Brain Institute, Mount Sinai School of Medicine, New York, New York, United States of America; 9 Fishberg Department of Neuroscience, Mount Sinai School of Medicine, New York, New York, United States of America; Hangzhou Normal University, China

## Abstract

The deprivation of sensory input after hearing damage results in functional reorganization of the brain including cross-modal plasticity in the sensory cortex and changes in cognitive processing. However, it remains unclear whether partial deprivation from unilateral auditory loss (UHL) would similarly affect the neural circuitry of cognitive processes in addition to the functional organization of sensory cortex. Here, we used resting-state functional magnetic resonance imaging to investigate intrinsic activity in 34 participants with UHL from acoustic neuroma in comparison with 22 matched normal controls. In sensory regions, we found decreased regional homogeneity (ReHo) in the bilateral calcarine cortices in UHL. However, there was an increase of ReHo in the right anterior insular cortex (rAI), the key node of cognitive control network (CCN) and multimodal sensory integration, as well as in the left parahippocampal cortex (lPHC), a key node in the default mode network (DMN). Moreover, seed-based resting–state functional connectivity analysis showed an enhanced relationship between rAI and several key regions of the DMN. Meanwhile, lPHC showed more negative relationship with components in the CCN and greater positive relationship in the DMN. Such reorganizations of functional connectivity within the DMN and between the DMN and CCN were confirmed by a graph theory analysis. These results suggest that unilateral sensory input damage not only alters the activity of the sensory areas but also reshapes the regional and circuit functional organization of the cognitive control network.

## Introduction

The functional organization of brain continues to change after prenatal development and can undergo remodeling throughout a person’s life to adapt to changing sensory experiences [1], [2]. Previous research has demonstrated that non-auditory sensory stimulation could activate the regions responsible for auditory processing in deaf people [3]–[5], which implies the existence of cross-modal plasticity in the sensory cortex of those who are completely deaf [6]. In addition, studies on the aging and cochlear implant populations [7]–[10] have demonstrated that hearing loss can alter different aspects of human cognitive functions in both pre- and post-lingual deafness [11]–[13], such as enhanced peripheral visual attention across time or space [14]–[17], behavioral inhibition defects [9], [18], distributed short-term memory [19] and compromised executive function [20]–[22]. Thus, there are at least two important forms of remodeling of the brain during hearing damage: the cross-modal plasticity implies that functional reorganization in sensory regions compensate for the compromised hearing input by optimizing multi-sensory perception [6] and changes in cognitive function suggest that more cognitive resources must be engaged during auditory processing to compensate for hearing impairment [11], [19].

Unlike those with bilateral deafness, unilateral hearing loss (UHL) individuals preserve much of the ability to capture auditory information, and the changes in auditory processing are more complicated [23]–[26]. Moreover, the brain is organized into intrinsic networks, which work cooperatively to facilitate an individual’s responses to stimuli [27], and these networks instantiate the maintenance of information for interpreting, responding to and even predicting environmental demands [28]. When one of the sensory perceptions is compromised, such as the asymmetrical auditory processing in UHL, it may affect not only the integral auditory perception [29] but also the auditory processing for higher-order representations [30]–[32]. It is therefore reasonable to predicate that the internal connectivity within sensory and high-order control networks as well as integration between these networks might be reorganized in UHL patients functionally. Previous research has addressed the issue of how UHL affects the plasticity in central auditory pathway, however, most of these studies are confined to the auditory cortex by examining task-related brain activation [33]–[36]. No study to date has investigated the functional changes associated with asymmetrical hearing damage and considered the reorganizations related to both sensation and cognitive functions. It remains unclear whether the deprivation of unilateral auditory input would affect the neural circuitry of the cognitive control network in addition to sensory cortex.

Resting-state fMRI is a promising noninvasive technique for mapping whole brain functional activity. Previous studies have demonstrated that regional homogeneity (ReHo) [37], a robust index with high test-retest reliability [38] can be used to map the synchronization of time courses between neighboring regions, as well as resting state functional connectivity (RSFC) [39] with excellent test-retest reliability [40] for measuring the temporal relationship among spatially distant regions, were quite informative for investigating the neural basis of individual differences in sensory deprivation [41]–[43] [32], [44], [45]. Moreover, resting-state fMRI is especially useful in clinical populations which have difficulty in task performances [27] such as patients with UHL, and it could provide functional information for simultaneously investigating the mechanisms of auditory and non-auditory higher order functional changes underlying UHL.

We hypothesized that the sensory cortex as well as regions subserving the higher order control network would be the primary regions affected by reduced hearing in UHL. We collected resting-state fMRI data from participants with UHL that resulted from acoustic neuroma and compared them to healthy controls to examine the plastic changes in regional homogeneity and functional connectivity in UHL. We predict that UHL patients would show abnormal resting activity not only in primary sensory regions, but also in the networks involving in cognitive control.

## Materials and Methods

### Participants

All participants were right-handed and reported no previous or current psychiatric disorders (see Table 1 for all participants’ demographic and clinical characteristics). Informed consent was obtained from all subjects prior to their participation, and all participants provided their written informed consent to participate in this study. The study was approved by the Institutional Review Board of Beijing Tiantan Hospital, Capital Medical University.

**Table 1:** Demographic and auditory characteristics of the participant groups.

	Left UHL	Right UHL	NC	*F* value	*p* value
Age (years)	45.7±6.5	43.0±5.4	46.0±4.8	0.337	0.715
Gender	12f/5m	8f/9m	13f/9m	1.945*	0.378
Handness	R	R	R	NA	NA
Education (years)	12.4±1.9	11.6±2.3	13.7±1.2	1.565	0.219
MMSE	26.8±1.2	27.5±1.1	28.1±0.6	2.033	0.141
Left ears PTA (dB)	67.2±19.2	18.6±3.8	16.0±2.2	1.925a	0.062
				−5.9^b^	0.000
Right ears PTA (dB)	19.1±2.3	66.8±15.6	15.6±2.4	1.272^c^	0.211
				−7.2^d^	0.000
				0.034^e^	0.973
Duration of UHL (months)	26.1±10.9	22.6±11.7	NA	0.437**	0.665

Abbreviations: UHL = unilateral hearing loss patients, MMSE = Mini-Mental Status Examination, f = female, m = male, R = right, NC = normal controls. Left and Right PTA = pure tone audiometry results of [0.5 kHz +1 kHz +2 kHz +4 kHz]/4 of the left and right ears,

*: *χ^2^* value of Chi-square test,

**: *t* value of t-test between duration of left and right UHL,

a : t value of t-test between PTA in left ears of right UHL and NC,

b : t value of t-test between PTA in left ears of left UHL and NC,

c : t value of t-test between PTA in right ears of left UHL and NC,

d : t value of t-test between PTA in right ears of right UHL of NC,

e : t value of t-test between PTA in impaired ears of left and right UHL,

Thirty-six untreated UHL patients with primary ipsilateral acoustic neuroma (AN) and 24 normal controls (NCs) participated in the study. Two UHL participants (One left UHL and one right UHL) and 2 NCs were excluded due to excessive head motion (please refer to Data Preprocessing). Finally, 34 UHL patients and 22 NCs were included in the study. Among the patients, 17 had hearing loss in the left ear and 17 had hearing loss in the right ear. NC were recruited from local communities in Beijing.

### Cognitive and Clinical Assessments

The cognition of all participants was evaluated by the Mini-Mental State Examination (MMSE) [46], which has been widely used clinically for screening cognitive impairment. It includes 11 items that assess eight categories of cognition: orientation to time, orientation to place, registration, attention and calculation, recall, language, repetition, and complex commands. The full score is 30 points and a low score indicates cognitive impairment. MMSE evaluation was performed to provide a brief and accurate report of the neurological status including mental status, cranial nerves, motor, sensory, coordination, and reflex functions of all patients.

Pure tone audiometry (PTA) is a gold standard for the assessment of hearing loss. We used the standard Hughson-Westlake PTA procedure to identify hearing threshold level of both ears for all participants in the study. The air-conduction pure-tone audiogram was assessed by a clinical audiologist under standard conditions. Audiometric measurements were performed using a GSI-61 audiometer, including TDH39 headphones. Audiologic equipment was calibrated on a regular basis. Pure tone audiometry was conducted at frequencies of 0.25, 0.5, 1, 2, 4, and 8 kHz. Acoustic thresholds of the affected ears were compared with those of the contralateral (unaffected) ears at each frequency level. The mean acoustic thresholds in speech frequency ([0.5 kHz +1 kHz +2 kHz +4 kHz]/4) were calculated to estimate the level of hearing loss.

### Demographic and Auditory Profile

Based on the evaluations of MMSE and standard neurological examinations, all the participants did not show cognitive, language, or somatosensory deficit besides hearing loss. As shown in Table 1, there was no significant group difference in age (*F* = 0.338, *p* = 0.715), gender (χ^2^ = 1.945, *p* = 0.378), education (*F* = 1.565, *p* = 0.219) or MMSE (*F* = 2.033, *p* = 0.141) among three groups of subject. Most of the UHL participants had severe-to-profound hearing loss in the impaired ear (eleven left UHL with PTA ≥50 dB and the other six 50>PTA≥20 dB; twelve right UHL with PTA ≥50 dB and the other five 50>PTA≥20 dB). UHL patients showed higher PTA levels of the impaired ear than those of the same side of ears in NCs (*t* = −5.9, *p*<0.001 for left UHLs; *t* = −7.2, *p*<0.001 for right UHLs). Meanwhile, no significant difference was found between PTA levels of unaffected ears in UHL patients and those of the same side of ears in normal controls (*t* = 1.272, *p* = 0.211 for left UHL; *t* = 1.925, *p* = 0.062 for right UHL). There was no significant differences in the duration of hearing loss (*t* = 0.437, *p* = 0.665), PTA between the left and right ears that were compromised (*t* = 0.034, *p* = 0.973), or the contralateral ears with intact hearing (*t* = −0.257, *p* = 0.799) between these two patient groups**.**

### Imaging Acquisition

All functional and structural images were acquired on a 3.0 Tesla scanner (Siemens Trio, Erlangen, Germany) using 12-channel head coil. Head movement was minimized using foam pads, and earplugs were used to attenuate acoustic noise during scanning. For the ten-minute resting-state fMRI scan, participants were instructed to hold still and keep their eyes closed, but not to fall asleep nor think of anything in particular. Resting-state fMRI data were acquired using an echo-planar image pulse sequence (41 axial slices, slice thickness/gap = 3.5/0.7 mm, repetition time = 2500 ms, echo time = 30 ms, flip angle = 90°, and field of view (FOV) = 256×256 mm^2^ with in-plane resolution of 3.75×3.75 mm^2^). A T1-weighted sagittal anatomical image was also obtained using a gradient echo sequence (176 slices, slice thickness/gap = 1/0 mm, inversion time = 900 ms, repetition time = 2300 ms, echo time = 3 ms, flip angle = 7°, number of excitations = 1, FOV = 256×256 mm^2^ with in-plane resolution of 0.9375×0.9375 mm^2^).

### Data Preprocessing

The resting-state fMRI data were preprocessed using SPM8 (http://www.fil.ion.ucl.ac.uk/spm ) and a pipeline analysis toolbox, DPARSF [47] http://www.restfmri.net/). To avoid transient signal changes before the longitudinal magnetization reached a steady state, the first ten volumes were discarded. The remaining images were preprocessed using a procedure, which included slice timing correction, head motion correction, T1-weighted image based spatial normalization to the Montreal Neurological Institute (MNI) space, linear trend removal, and band-pass filtering (0.01–0.08 Hz). All of the participants’ head motion parameters were less than 3 mm in translation and less than 3 degrees in rotation. To further reduce the effects of head motion on estimates of resting-state activity, we censored volumes within each participant’s fMRI time series that were associated with sudden head motion [48], [49]. For each participant, fMRI volumes were censored if framewise displacement (FD) of head position, calculated as the sum of the absolute values of the derivatives of the realignment estimates, was above 0.5. As a result, four participants (one left UHL, one right UHL and two NCs) with less than five minutes of data after “scrubbing” were excluded from the further analysis.

### Data Length and Mean FD after “scrubbing”

The mean numbers of volumes of left UHLs, right UHLs and NCs after scrubbing were 229.9, 224.5 and 226.2, respectively. There waere no significant differences in the number of volumes between the three groups of subjects (*p* = 0.77). Mean FD of the three groups after scrubbing were 0.11 mm, 0.12 mm and 0.13 mm, respectively. No significant statistical difference was found between mean FD of the three groups of subjects (*p* = 0.68).

### ReHo Analysis

ReHo [37] was used to measure local synchronization of spontaneous BOLD fluctuations within a given cluster (e.g. 27 nearest neighboring voxels). Kendall’s coefficient of concordance (ranged from 0 to 1) was used as a measurement of ReHo for each voxel, an indication of similarity between the time series of that voxel and its nearest neighboring voxels. It was measured in a voxel-wise way for each participant within a whole brain mask provided by REST [50]. To reduce nuisance sources of variation [51], individual ReHo maps were divided by the global mean value within the whole brain mask for normalization. Then, all normalized ReHo maps were spatially smoothed with a 6-mm full width at half-maximum (FWHM) Gaussian isotropic kernel.

To detect differences in ReHo amongst the three groups of participants, a one-way analysis of covariance (ANCOVA) was conducted based on the ReHo maps within the whole brain mask with age and gender information as covariates. The Gaussian Random Field theory, which has been implemented in REST, was used to correct for multiple comparisons. The corrected *p*<0.05 (uncorrected *p*<0.001 and minimum 21 voxels in a cluster) was used as threshold. To determine which pairs of groups contributed to the significant group difference, post hoc Tukey pairwise comparison was conducted between each pair of groups for each cluster, with a significance threshold set at 0.05.

### Seed-based Resting-state Functional Connectivity Analysis

Significant clusters were extracted from ANCOVA of ReHo maps and served as seeds in resting-state functional connectivity analysis. First, non-neuronal-related covariates, including six parameters of head motion correction, the average time courses of the whole brain (global mean signal), the average time courses within the white matter mask, and the average time courses within the CSF mask, were removed from the preprocessed data by linear regression analysis. Then, the images were spatially smoothed with a 6-mm FWHM Gaussian kernel. We computed the functional connectivity between each seed region and every voxel within the whole brain mask. The individual functional connectivity maps were transformed to *z*-maps using Fisher’s *z*-transformation to improve data normality. A series of one-sample *t*-tests were conducted to detect cortical areas functionally connected with each seed region in each group of participants (Figs. S2–S3). A group connectivity mask was generated with an “OR” operation of the corrected functional connectivity maps of the three groups for each seed. For each seed region, one-way ANCOVA, with age and gender as covariates, was used to identify brain regions within the mask, with significant differences in connectivity to the seed region among the three groups. Multiple comparison correction was performed according to the Gaussian Random Field theory with a corrected *p*<0.05 (uncorrected *p*<0.001 and minimum 21 voxels in a cluster) within the whole brain mask. Similar to ReHo analysis, post hoc Tukey pairwise comparisons were conducted between each pair of groups for each significant cluster identified by the ANCOVA of seed-based functional connectivity.

Further, to examine more directly the potential reorganization of brain activity in the default mode network (DMN) and cognitive control networks (CCN) subserving higher-order cognitive functions, we performed ROI-based functional connectivity analysis using previously defined ROIs (Table S1) of the DMN and cinguloopercular network, a representative networks that compose the CCN involving rAI.

### Correlation between Intrinsic Resting-state Activity and Degree and Duration of Hearing Loss

To investigate the relationship between brain plasticity and PTA as well as hearing loss duration in participants with UHL, we calculated the correlation between the above computed resting-state activity indices (i.e. ReHo/RSFC) and PTA and hearing loss duration. Corrected *p*<0.05 was used as threshold for multiple comparisons correction (uncorrected *p*<0.01, corrections for ReHo and for RSFC analyses were performed using the whole brain mask and the Group connectivity mask, respectively).

## Results

### Abnormal Regional Activity of UHL Patients in ReHo

**Figure 1:**
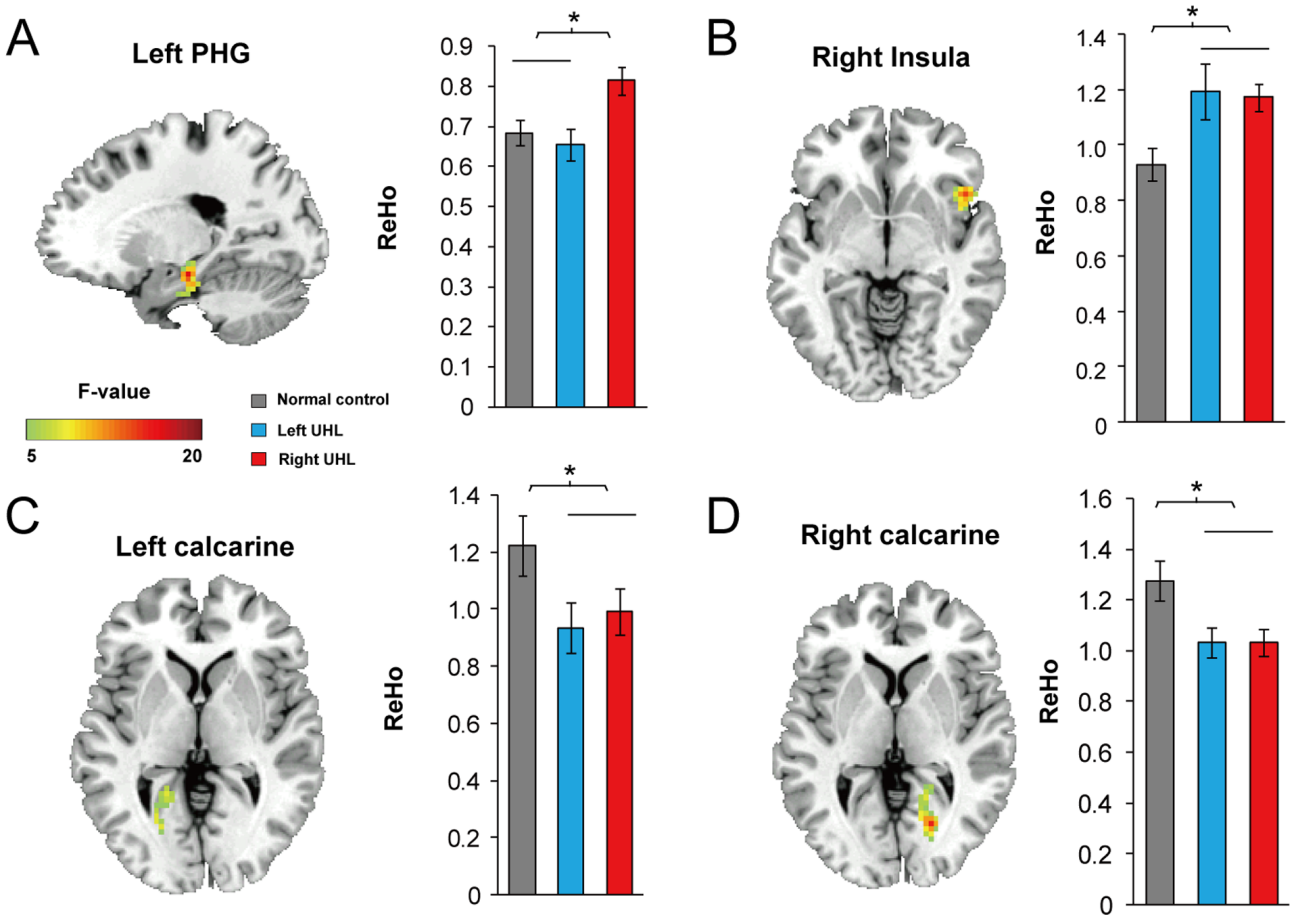
Group difference in ReHo revealed by ANCOVA. Significant differences, location and ReHo values of each group in right anterior insular cortex (rAI) (A), left parahippocampal cortex (lPHC) (B), left calcarine cortex (C) and right calcarine cortex (D). The bar and error bar represent the mean value and SD, respectively, of the ReHo values in the region. * *p*<0.05, corrected. UHL, unilateral hearing loss, NC, normal controls.

The ANOVA of ReHo showed significant group differences in cortical regions, including left parahippocampal cortex (lPHC), right anterior insular cortex (rAI), and bilateral calcarine cortices (see Fig. 1), indicating that the neural synchronization of local brain areas during resting state were reshaped by auditory deprivation in UHL. Further post hoc comparisons amongst the lPHC, rAI and bilateral calcarine cortices (Fig. 1, see Table 2 for details, *p*<0.05) showed that left UHL and right UHL participants had higher ReHo values in the rAI compared to NCs (post-hoc *p*<0.001) (Fig. 1A). Right UHL participants showed higher ReHo values than NC and left UHL participants (post-hoc *p*<0.001) in the left PHC (Fig. 1B). Meanwhile, left and right UHL participants had lower ReHo in bilateral calcarine cortices compared to NCs (post-hoc *p*<0.001) (Fig. 1C–1D).

**Table 2:** Group difference in ReHo among the three groups of participants.

Location	Peak	MNI coordinate (mm)			Volume	Post hoc analysis
	F-value	x	y	z	(mm^3^)	(Tukey *p*<0.05)
right AI	15.41	48	18	−6	39	Left UHL and Right UHL>NC
left PHC	17.22	−21	−21	−18	46	Right UHL>Left UHL and NC
left calcarine cortex	11.87	−21	−48	−3	43	Left UHL and Right UHL<NC
Right calcarine cortex	16.00	21	−69	3	77	Left UHL and Right UHL<NC

Abbreviations: AI = anterior insular cortex, PHC = parahippocampal cortex, UHL = unilateral hearing loss, NC = normal controls.

### Abnormal Circuit Activity of UHL Patients within and Beyond the Auditory System

**Figure 2:**
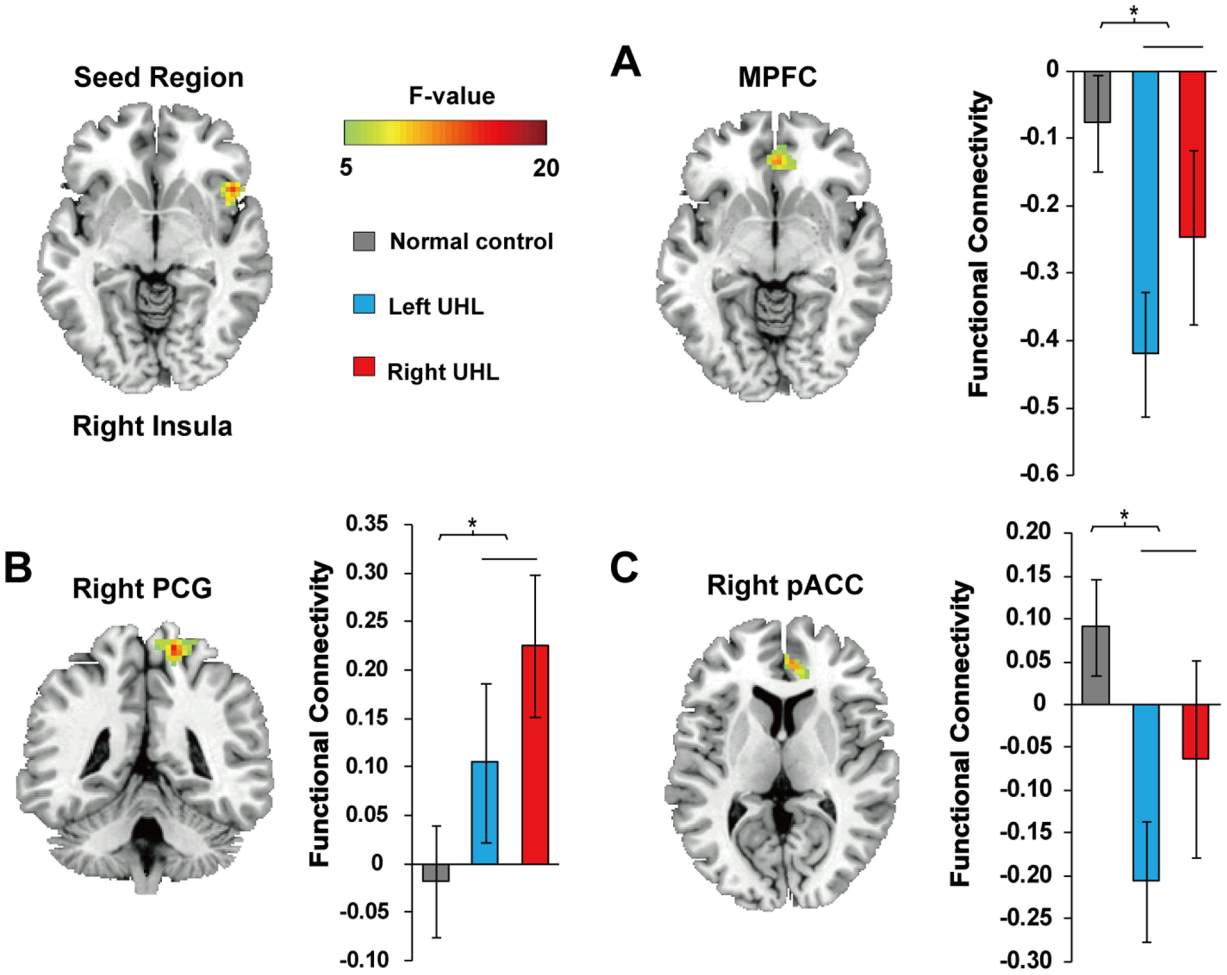
Group difference in resting state functional connectivity with rAI (the seed region) revealed by ANCOVA. The significant differences were shown in right medial prefrontal cortex (rMPFC) (A), right postcentral gyrus (rPCG) (B) and pregenual anterior cingulate cortex (pACC) (C). The bar and error bar represent the mean value and SD, respectively, of the functional connectivity values in the region. * *p*<0.05, corrected.

**Figure 3:**
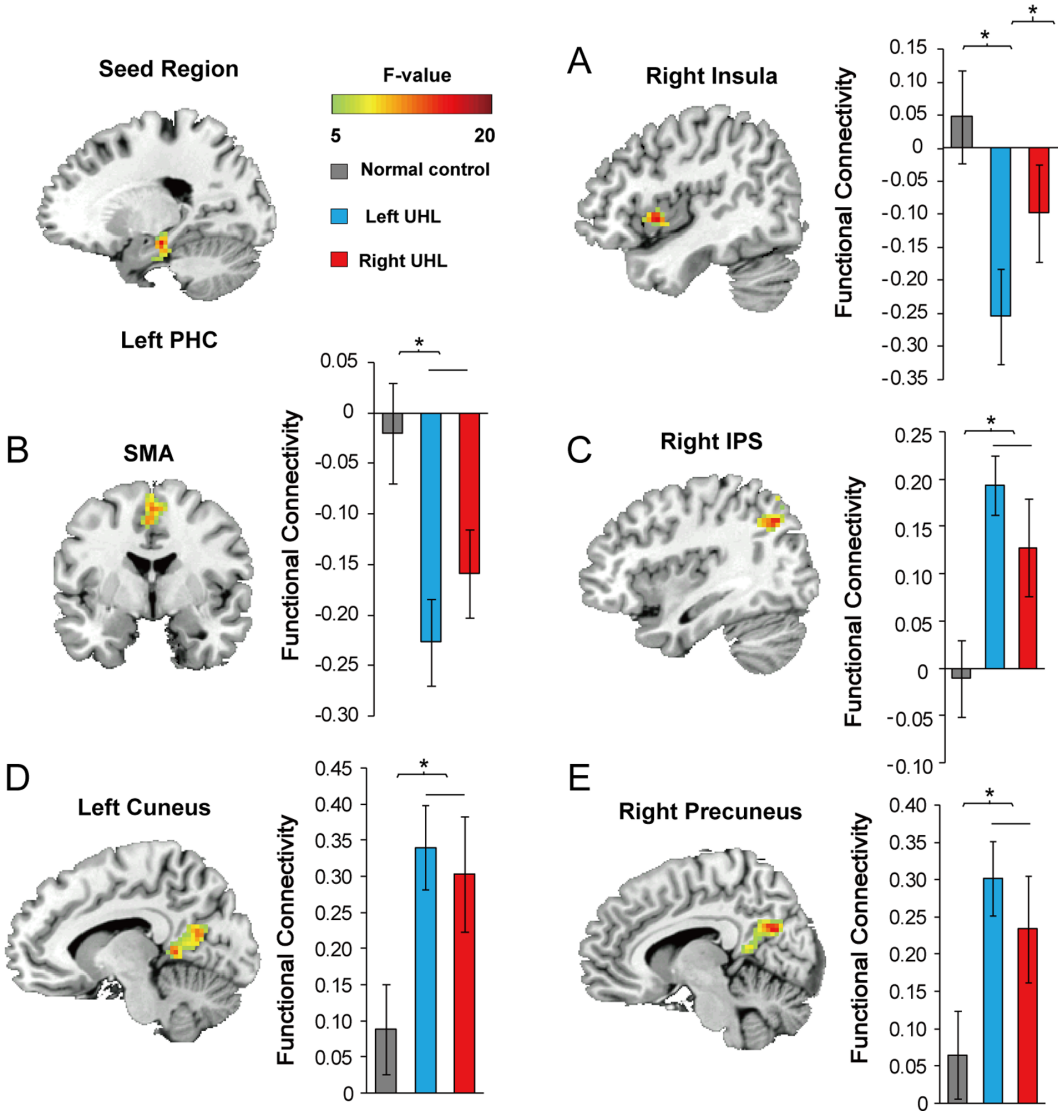
Group difference in resting state functional connectivity with lPHC (seed region) revealed by ANCOVA. The significant differences are shown in (A) right insula, (B) supplementary motor area (SMA), (C) right angular gyrus (rAG), (D) left cuneus and (E) right precuneus. The bar and error bar represent the mean value and SD, respectively, of the functional connectivity values in the region. * *p*<0.05, corrected.

Four regions identified from ReHo analyses were used as seeds for RSFC analysis, including lPHC, rAI and bilateral calcarine cortices, respectively. ANCOVA on the functional connectivity of the rAI seed region showed significant group differences in the medial prefrontal cortex (rMPFC), right pregenual anterior cingulate cortex (pACC) and right postcentral gyrus (rPCG) (post-hoc *p*<0.05, see Fig. 2). Using lPHC as a seed, DMN regions (i.e. right angular gyrus, right precuneus, left cuneus) showed the stronger connectivity among UHL patients compared with NC (post-hoc *p*<0.05, see Fig. 3). There was no significant difference among three groups in RSFC with bilateral calcarine cortices as the seeds. Post-hoc pairwise comparisons (see Table 3 for details, *p*<0.05) showed increased strength of functional connectivity in UHL patients compared to normal controls by using lPHC and rAI as seeds, while no findings of decreased RSFC strength in UHL. Importantly, most of these regions showing altered RSFC were associated with higher-order brain structures including those of the DMN (lPHC, left cuneus, right precuneus and MPFC) and CCN (rAI, rACC), and these results were confirmed by a graph theory analysis (see Text S1, Fig. S6 and Fig. S7).

**Table 3:** Group difference in functional connectivity with seed regions selected from ReHo analysis.

Seed	Location	Peak	MNI coordinate (mm)			Volume	Post hoc analysis
		F-value	x	y	z	(mm^3^)	(Tukey *p*<0.05)
	MPFC	13.46	3	39	−6	48	Right UHL and Left UHL< NC <0
Right AI	right pACC	13.30	6	45	9	39	Right UHL and Left UHL <0< NC
	right PCG	17.94	15	−45	69	89	Right UHL and Left UHL >0> NC
	right AI	17.26	48	9	−3	76	Left UHL< Right UHL<NC <0
	SMA	15.02	0	9	51	121	Right UHL and Left UHL< NC <0
Left PHC	Right AG	18.77	36	−69	39	227	Right UHL and Left UHL >0> NC
	Left cuneus	14.95	−9	−48	3	139	Right UHL and Left UHL>NC >0
	Right precuneus	16.27	9	−66	24	123	Right UHL and Left UHL>NC >0

Abbreviations: AI = anterior insular cortex, PHC = parahippocampal cortex, MPFC = medial prefrontal cortex, pACC = pregenual anterior cingulate cortex, PCG = postcentral gyrus, SMA = supplementary motor area, AG = angular gyrus, UHL = unilateral hearing loss, NC = normal controls.

To note, neither ReHo nor RSFC showed a significant correlation with hearing loss duration or auditory ratings.

## Discussion

Exploring intrinsic brain activity is important for the understanding of brain organization and it may best capture the essence of brain function [55]. In current study, we found decreased ReHo in bilateral calcarine cortices in UHL patients. Moreover, ReHo was increased in the rAI, the key node of CCN, and in the lPHC, a key node of DMN, while the reorganization of RSFC within DMN and between DMN and CCN was confirmed by graph theory analysis. These findings support the notion that adaptive sensory-driven plasticity is involved in widespread brain areas during unilateral hearing processing. The UHL not only reshapes the activity of the sensory cortex but also alters the regional and circuit functional organization of the higher order control networks.

### Plasticity of Sensory Cortices

As ReHo during resting state might be the foundation of the activity change during task-state [56], the decreased ReHo of bilateral calcarine cortices in UHL compared with controls may present the baseline abnormality of sensory cortices in UHL during resting state. The calcarine cortex processes visual information and is considered to be the primary visual cortex. Decreased ReHo of the bilateral calcarine cortices in UHL groups compared with controls may represent an adaptation to engage other sensory systems to compensate for the partial loss of hearing [57]. It has been suggested that sensory deprivation in one modality (auditory) could affect the functions of the remaining modalities (visual) [6], [58] [59]. Previous research of auditory brain plasticity on congenitally deaf individuals suggested that early deafness is associated with activation and hyper-functionality in the visual cortex when having auditory stimulation [58], [60], which implies cross-modal plasticity. In the current study, all the hearing damage participants were post-lingual UHL adults, and they were not in the critical onset of age for cortical development. Therefore, the progressive UHL caused by AN may influence the calcarine cortex and induce reorganization of visual processing. The reduced ReHo in calcarine cortex may suggest the local reorganization in visual areas. The progressive UHL disrupted the integrity of sensory perceptions, and the visual cortex might not able to reorganize promptly but be in the process of remodeling showing as the reduced functional coherences. We inferred that the decreased ReHo may reflect the functional differentiation for better visual perception in order to cover the hearing deficits. To not, however, this does not mean a causal relationship between decreased ReHo and dysfunction of the visual region. Moreover, we did not find a significant difference in RSFC of bilateral calcarine cortex amongst three groups in current study. We propose that this may be due to the fact that UHL participants largely preserve the ability to capture auditory information in the unaffected ear. UHL may only cause local functional changes in visual areas, rather than long distance reorganization related to cross-model plasticity.

It is worth noting that, the auditory cortex is expected to be reorganized during UHL intuitively. In current study, attenuation of hearing input leads to a functional in-coordination between auditory perception and goal-directed attention as long as we assess the RSFC for auditory areas particularly (see Text S1,Table S2, Fig, S4 and Fig. S5). However, The UHL patients in current study were all postlingual deaf and their hearing loss started after the critical onset for cortical development. In addition, the UHL individuals did not suffer from totally deafness for a long enough duration. Previous study proved that the age of the onset of deafness is critical to plasticity of deaf brain[41]. Therefore, we could only find the impact on the auditory areas of UHL showing as a trend of alteration of ReHo and RSFC in regions related to auditory function such as temporal cortex (Fig. S1, Fig. S2 and Fig. S3). Nevertheless, the plastic changes in auditory cortex is too mild to pass the strict multiple-comparisons correction of ANOVA. The intrinsic activity changes within auditory cortex of UHL may be little but complex which needs to investigated by further studies.

### Functional Reorganization in the Higher Order Control Network

In this study, key nodes in the functional networks of CCN and DMN, such as rAI, lPHC, rather than primary sensory areas demonstrated both abnormal regional and circuit activity/connectivity in UHL, supporting our hypothesis that compensatory remodeling of higher order cognitive processes was involved in asymmetric auditory impairment.

#### Remodeling of the CCN

In the current study, rAI was demonstrated to show increased ReHo, increased strength of negative RSFC with pACC and MPFC in UHL participants compared to control subjects, supporting the notion that UHL lead to hyperactivity of rAI with local and long-distance plastic changes, which may suggest remodeling in cognitive control regions necessary for auditory processing of unilateral hearing damage.

It is recognized that complex auditory information processing may be hindered by UHL, such as phonological processing, musical perception, vocal communication sounds, and spatial and temporal auditory processing [23]–[26]. Virtually, all these high-level auditory processes rely on the cognitive control and conditioning of cognitive process [61], [62]. The AI plays an important role in several independent but interrelated CCN simultaneously [53], [63], [64]. For instance, AI serves in the cinguloopercular network which contribute to flexible control of human goal-directed behavior [54], [65], [66], as well as in salience networks which are required for detecting and orienting to salient external stimuli and internal events [31], [67], [68]. These networks have been proposed to exhibit altered intrinsic activity or connectivity in the AI due to compromised cognitive control in a number of pathological situations [69], [70] [71] [68], [72], [73]. These features serve as the theoretical basis for the speculation that alterations of spontaneous fMRI BOLD signals in rAI might due to the harder cognitive control as a compensation to the partial hearing loss in UHL.

In addition, the insula is also a critical region for the integration of information from diverse functional systems. It connects functional networks together to make perceptual decisions and support complex behavior [67], [74]–[76]. The AI integrates bottom-up signaling with top-down predictions to generate current awareness state [77]–[81]. Moreover, the AI is also believed to be novelty-sensitive region for involuntary attention to events in the sensory environment [82], [83]. For UHL individuals, the asymmetrical auditory deficits is aberrant from the vast and continuous stream of normal bilateral auditory stimuli, which might engage the rAI mediating cognitive control as well as engage different cognitive processes to compensate the perceptional deficits.

Taken together, in line with the hypothesis that altered functional connectivity reflects the brain characteristic in hearing loss [11], [19], [84], we speculated that the hyperactivity of the rAI and its abnormal RSFC in UHL as an indication of the alteration in cognitive control, namely, that reorganization in UHL individuals tend to favor efforts to resume the effective perception by remodeling the cognitive processing. However, future fMRI studies employing cognitive tasks are needed to further clarify the specific modalities of such cognitive reorganization.

#### Alteration of regional and circuit resting-state activity in DMN

Further, we found increased ReHo in the lPHC as well as enhanced positive RSFC between the lPHC and right angular gyrus, right precuneus and left cuneus in UHL compared to control subjects, suggesting reorganization within the DMN. The DMN, with all the above described regions as important nodes [52], is a task-negative, cross- spatial and intrinsically organized network in the brain. Numerous studies have found that the DMN remains stable amongst healthy individuals [85], [86] and can be affected by neurological and psychiatric diseases [87], [88]. In healthy subjects, the DMN involved in the integration of self-monitoring, autobiographical, and related social cognitive functions [86], [89]. The anti-correlated interactions between cognitive-demanding regions and the DMN are intrinsic through the human brain, occurring naturally and spontaneously [86], and it is correlated to a more consistent behavioral performance [90] as well as exact control of efficient goal-directed behavior and focused attention [91]. Viewed from this context, the increased positive RSFC between core nodes in DMN, which consistent with the enhanced correlation among key nodes within DMN in ROI-based functional connectivity analysis, indicated hyperfunction of DMN in UHL participants, suggesting that UHL induced an alteration in the intrinsic circuit for DMN in UHL.

#### Reorganization of the connectivity between CCN and DMN

In addition, we found increased strength of negative RSFC between the important nodes of DMN (MPFC, rAG and lPHC) and rAI in UHL participants compared to their control counterparts. Enhanced connectivity between CCN and the DMN was also demonstrated by the graph theory analysis. The alteration in RSFC has been demonstrated due to changes in the different sensory experience [32], [44], [45], such as blindness. In this study, the enhanced RSFC between nodes of CCN and DMN may indicate the more rapid and intensive coordination between these two networks for assisting the detection of dynamic environment and compensating the perceptional deficit resulting from UHL [90]. Moreover, the rAI plays a critical and causal role in switching between activation and deactivation of large-scale brain networks involving the central-executive network and the DMN [92]. The enhanced synchronization between lPHC and rAI in UHL may also indicate a prompt switching from resting state to an activated state in order to facilitate the cognitive process to adapt to attenuation of hearing information input. It would be in line with the notion that neural synchrony between spatially distinct regions helps to coordinate information processing in those regions [93]. Thus, changes within and between functional networks during resting-state might be an indicator of abnormal task activation and deactivation patterns and task performances, which encourages further investigations using task-based fMRI.

### No Correlation between Intrinsic Resting-state Activity and Degree and Duration of Hearing Loss

Neither ReHo nor RSFC showed a significant correlation with hearing loss duration or auditory ratings. The null findings might be due to some possible reasons. Firstly, a previous study has suggested that the influence of plasticity may be more so influenced by the onset of (pre- or post-lingually) the patients acquires a profound hearing impairment rather than the duration of auditory deprivation [94]. Secondly, duration of hearing loss in the current study was reported by the patients potentially be with subjective bias, and the unit for duration of hearing loss (months) may not offer enough temporal resolution to detect correlations between hearing loss duration and resting-state brain activity. It is also possible that the correlations between resting-state activity and degree or duration of hearing loss may not be linear.

### The Lateralization of UHL

For most of the result, the UHL groups showed significant differences compared to normal control groups with non significant differences between left and right UHL groups. However, most reorganizations of ReHo and RSFC found in the current study were more pronounced in left UHL patients than right UHL ones, although not significant statistically. This may have resulted from the fact that greater resilience against reduced hearing damage in right ear [95]. The left ear pathway demonstrates a stronger contralateral effect during monaural acoustic stimulation [96], [97]. In other words, the right ear delivers information to both hemispheres more evenly, while the left ear delivers information more lateralized. Accordingly, once the normal pattern was interfered by UHL, the dysfunction of left ear interrupt the brain more significantly. Meanwhile, the right UHL participants show more stabilization in cortex.

## Conclusion

The present study confirms and extends the previous findings of reorganizations in the brain associated with auditory damage. The reduced regional homogeneity in calcarine cortex suggested a dysfunctional reorganization for post-lingual UHL patients in sensory cortex. Furthermore, both regional and circuit plastic changes in rAI and lPHC which involving in high-order cognitive networks may indicate the adapted cognitive process for attenuation of hearing information input. Taken together, abnormal resting-state brain activity in UHL participants suggests that brain areas involved in cognitive control as well as the sensory cortices, are modulated by asymmetric auditory function preservation.

## Supporting Information

Figure S1 One-sample t-maps of ReHo in the whole brain for each of the three groups (*p*<0.05, corrected). L and R represent the left and right hemispheres, respectively. The results were mapped onto the cortical surfaces using in-house developed BrainNet viewer software (www.nitrc.org/projects/bnv/). UHL, unilateral hearing loss, NC, normal controls.
(TIF)

Figure S2 One-sample t-maps of resting-state functional connectivity of the right anterior insular cortex in the whole brain for each of the three groups (*p*<0.05, corrected). L and R represent the left and right hemispheres, respectively. The results were mapped onto the cortical surfaces using in-house developed BrainNet viewer software (www.nitrc.org/projects/bnv/).
(TIF)

Figure S3 One-sample t-maps of resting-state functional connectivity of the left parahippocampal cortex in the whole brain for each of the three groups (*p*<0.05, corrected). L and R represent the left and right hemispheres, respectively. The results were mapped onto the cortical surfaces using in-house developed BrainNet viewer software (www.nitrc.org/projects/bnv/).
(TIF)

Figure S4 Group difference in resting state functional connectivity with left HG(the seed region) revealed by ANCOVA. The significant differences were shown in left medial frontal gyrus (MPG) (A) and right superior parietal lobule (SPL) (B). The bar and error bar represent the mean value and SD, respectively, of the functional connectivity values in the region. * *p*<0.05, corrected.
(TIF)

Figure S5 Group difference in resting state functional connectivity with right HG (the seed region) revealed by ANCOVA. The significant differences were shown in left medial frontal gyrus (MFG) (A). The bar and error bar represent the mean value and SD, respectively, of the functional connectivity values in the region. * *p*<0.05, corrected.
(TIF)

Figure S6 The distribution of connections with significant group effects in the functional connectivity strength among the three groups at *p*<0.01 (uncorrected). The thicknesss of connections indicate the significance of between-group differences. ROIs in purple belong to DMN while ROIs in blue belong to CCN. Connections in gray indicate those connections were between DMN and CCN (A–C), while connections in purple indicate those connections were within DMN [ipsilateral connetions (D–E), contralateral connections (F–J)]. For each connection, the bar and error bar represent the mean value and SD, respectively, of the functional connectivity strength in each group. Post hoc tests showed that all the ROIs have increased functional connectivity strength in the left UHL patients versus the controls. Three of these six ROI, including the rIPL, rTPJ and rAI/fO showed reduced functional connectivity strength in the right UHL patients compared with the left ones. Only one region (lHF) showed increased functional connectivity strength in right UHL patients compared to normal controls. * *p*<0.05. The connecitons were mapped onto the cortical surfaces using in-house BrainNet viewer software (www.nitrc.org/projects/bnv/).
(TIF)

Figure S7 The distribution of brain regions with significant group effects in the functional connectivity strength among the three groups at *p*<0.05 (uncorrected). The sizes of ROIs indicate the significance of between-group differences. ROIs in purple belong to DMN while ROIs in blue belong to CCN. For each ROI, the bar and error bar represent the mean value and SD, respectively, of the functional connectivity strength in each group. Post hoc tests showed that all the ROIs have increased functional connectivity strength in the left UHL patients versus the controls. Three of these six ROI, including the rIPL, rTPJ and rAI/fO showed reduced functional connectivity strength in the right UHL patients compared with the left ones. Only one region (lHF) showed increased functional connectivity strength in right UHL patients compared to normal controls. * *p*<0.05. The ROIs were mapped onto the cortical surfaces using in-house BrainNet viewer software (www.nitrc.org/projects/bnv/). For the abbreviations of the ROIs, see Table S1.
(TIF)

Table S1 Regions of interest in graph analysis.
(DOC)

Table S2 Group difference in functional connectivity with seed regions at primary auditory cortex.
(DOC)

Text S1 (DOC)
