# Supplementary figures and images for: Altered Regional and Circuit Resting-State Activity Associated with Unilateral Hearing Loss
Source: PLoS One. 2014 May 1;9(5):e96126. doi: 10.1371/journal.pone.0096126 (PMC4006821; doi:10.1371/journal.pone.0096126)

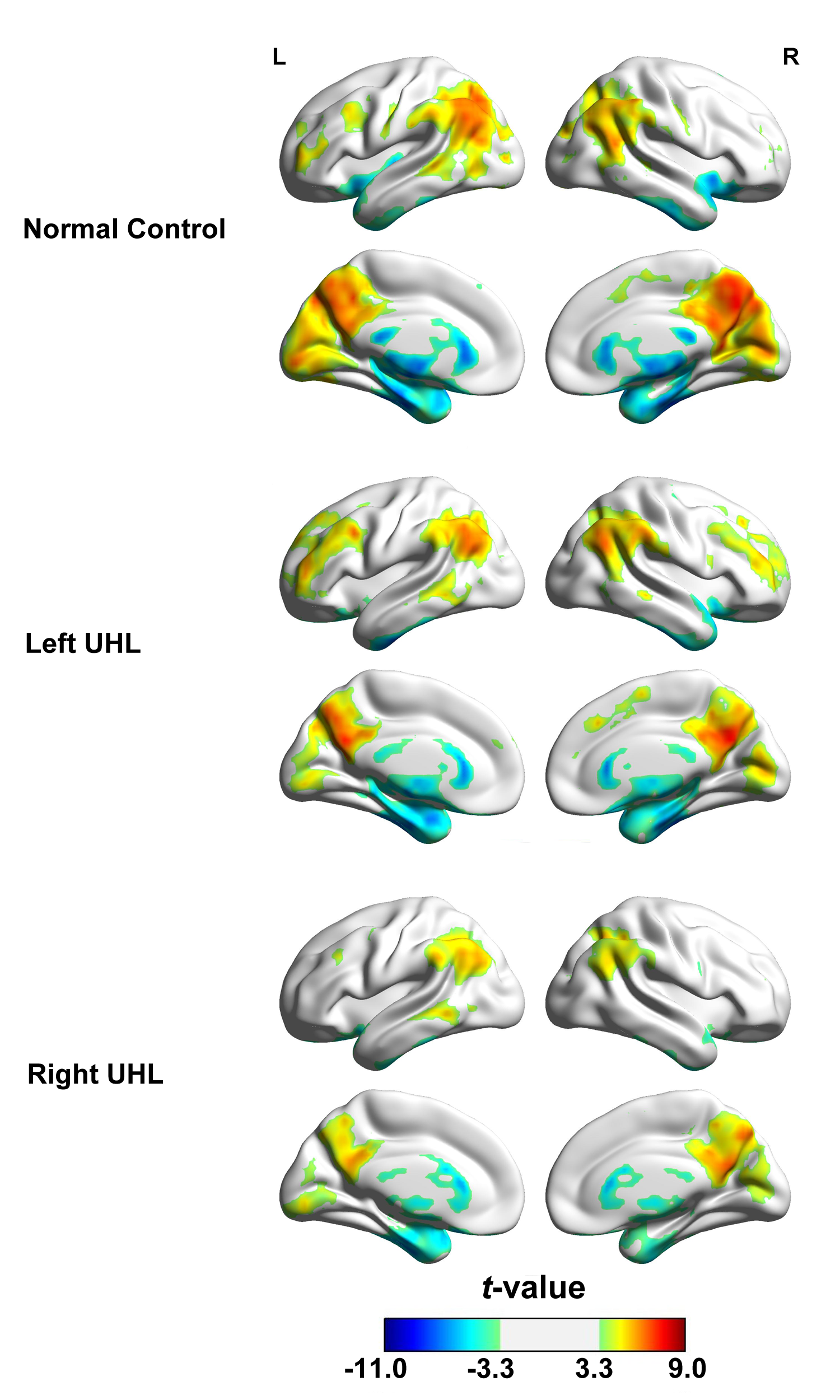

Supplement: Figure S1 — One-sample t-maps of ReHo in the whole brain for each of the three groups (p<0.05, corrected). L and R represent the left and right hemispheres, respectively. The results were mapped onto the cortical surfaces using in-house developed BrainNet viewer software (www.nitrc.org/projects/bnv/). UHL, unilateral hearing loss, NC, normal controls. (TIF) [file pone.0096126.s001.tif]

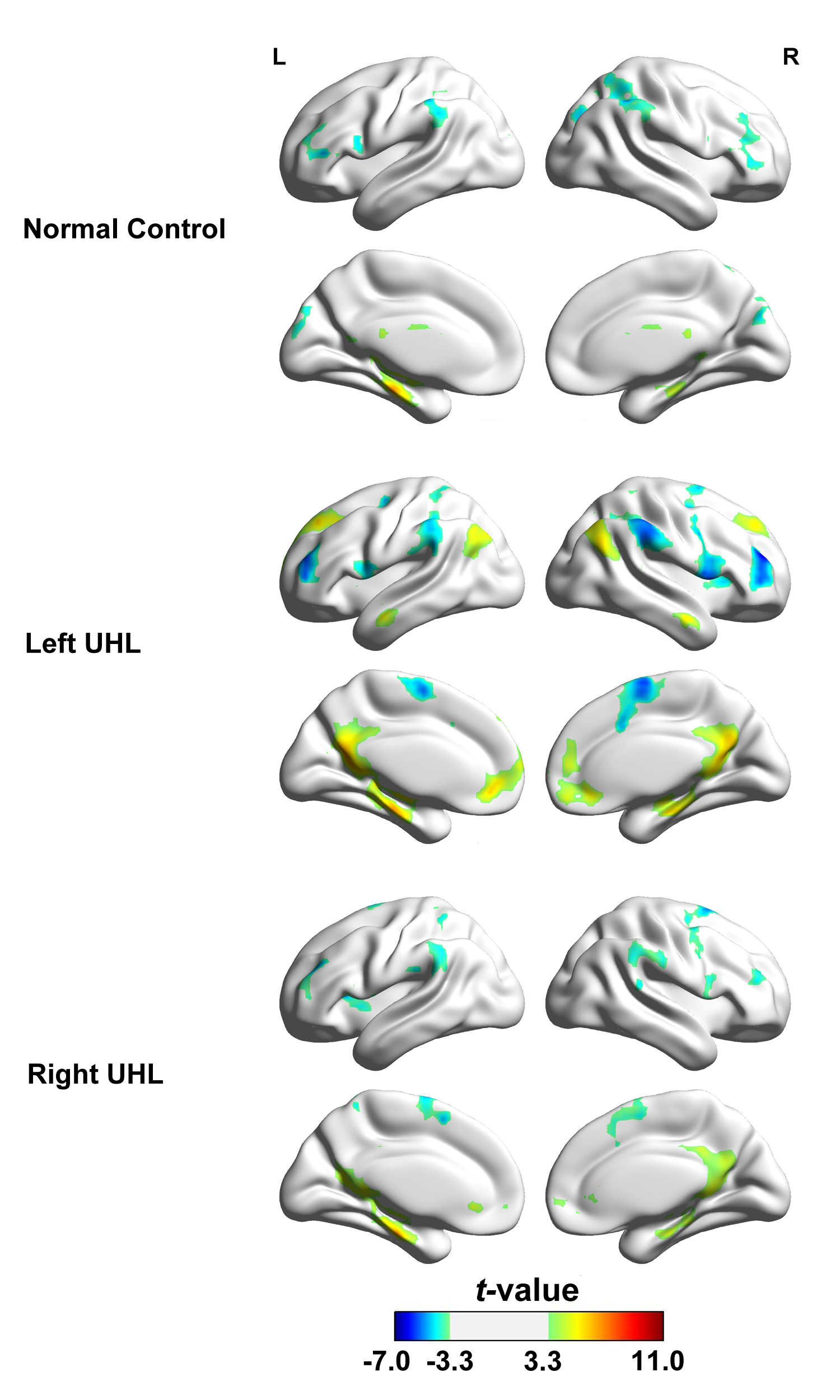

Supplement: Figure S2 — One-sample t-maps of resting-state functional connectivity of the right anterior insular cortex in the whole brain for each of the three groups (p<0.05, corrected). L and R represent the left and right hemispheres, respectively. The results were mapped onto the cortical surfaces using in-house developed BrainNet viewer software (www.nitrc.org/projects/bnv/). (TIF) [file pone.0096126.s002.tif]

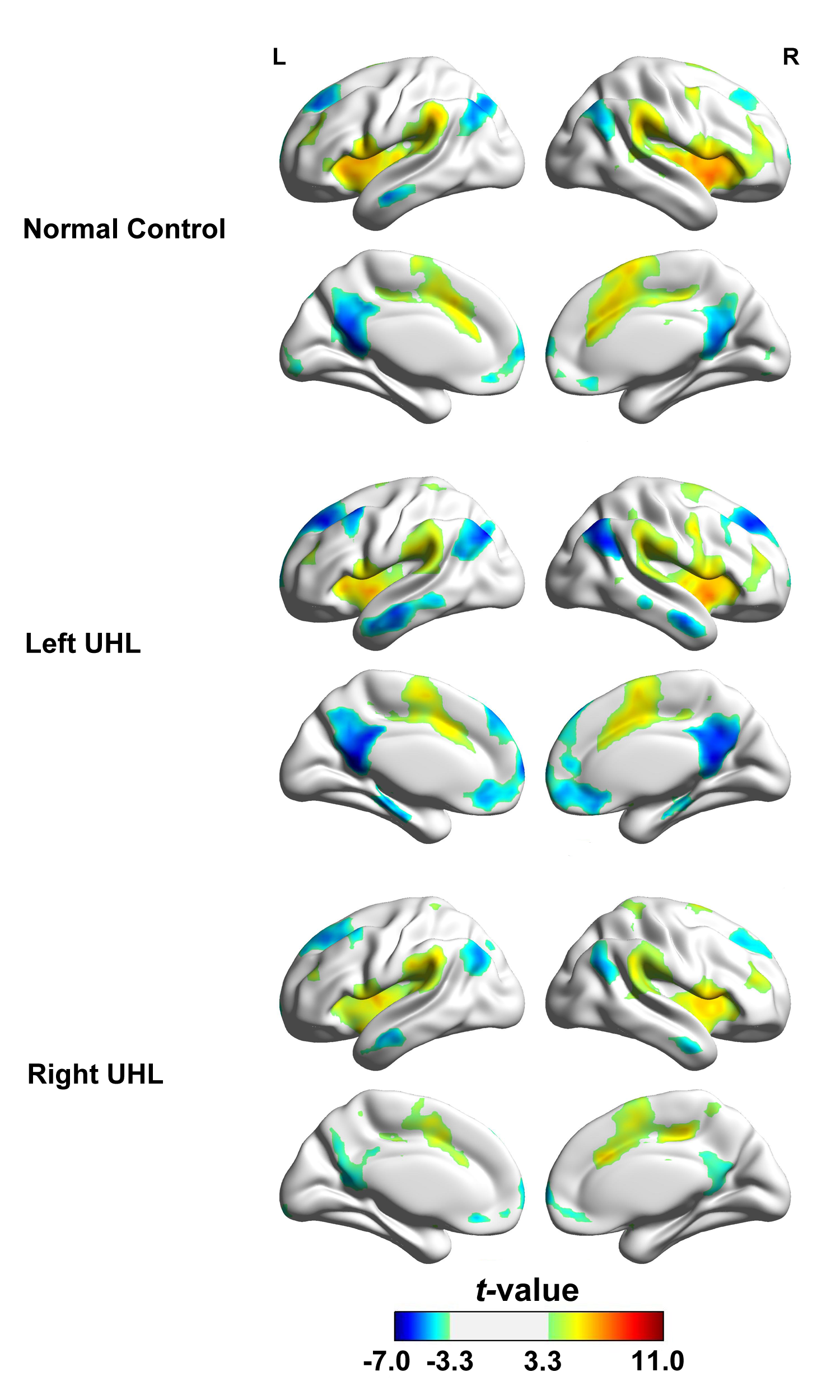

Supplement: Figure S3 — One-sample t-maps of resting-state functional connectivity of the left parahippocampal cortex in the whole brain for each of the three groups (p<0.05, corrected). L and R represent the left and right hemispheres, respectively. The results were mapped onto the cortical surfaces using in-house developed BrainNet viewer software (www.nitrc.org/projects/bnv/). (TIF) [file pone.0096126.s003.tif]

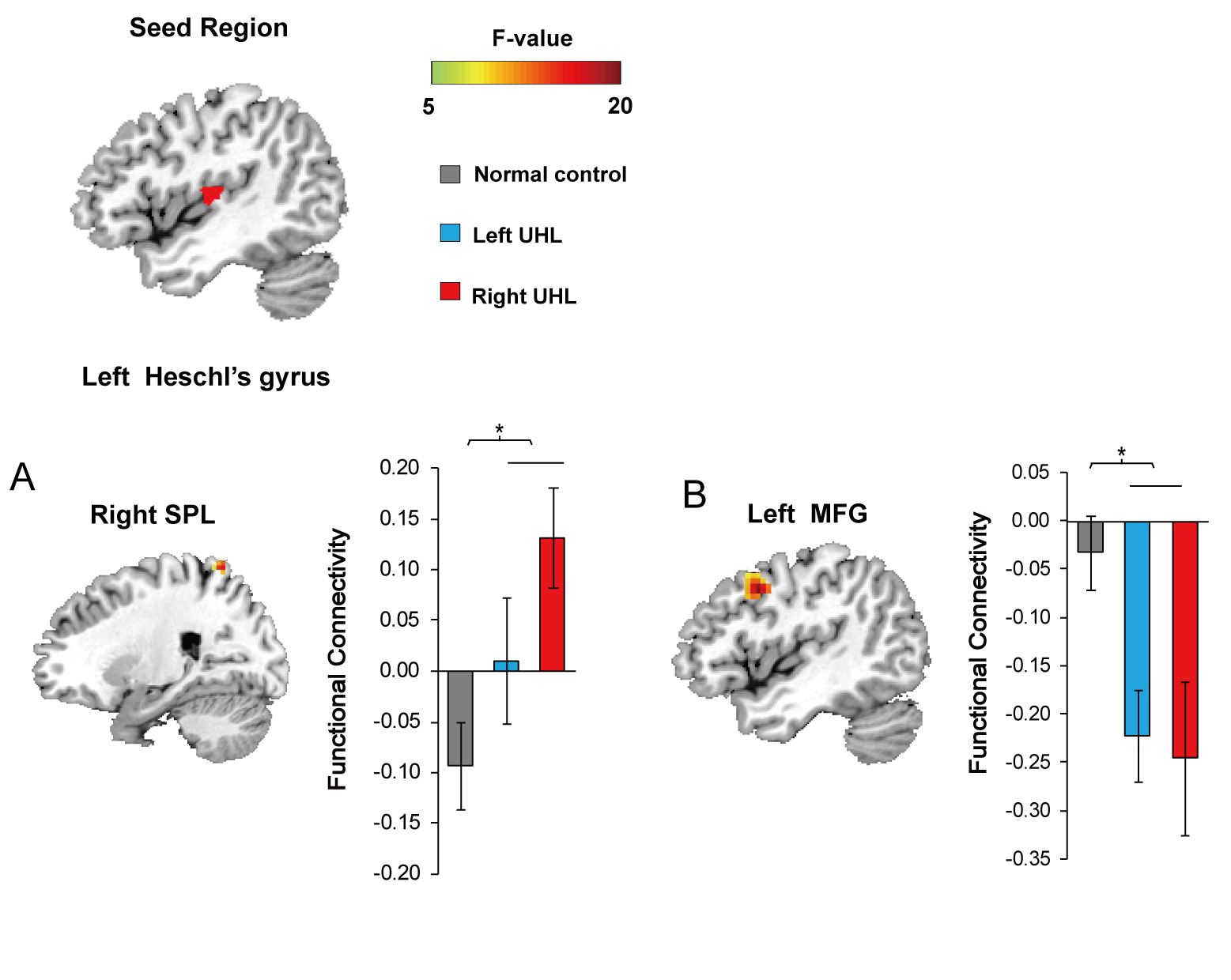

Supplement: Figure S4 — Group difference in resting state functional connectivity with left HG(the seed region) revealed by ANCOVA. The significant differences were shown in left medial frontal gyrus (MPG) (A) and right superior parietal lobule (SPL) (B). The bar and error bar represent the mean value and SD, respectively, of the functional connectivity values in the region. * p<0.05, corrected. (TIF) [file pone.0096126.s004.tif]

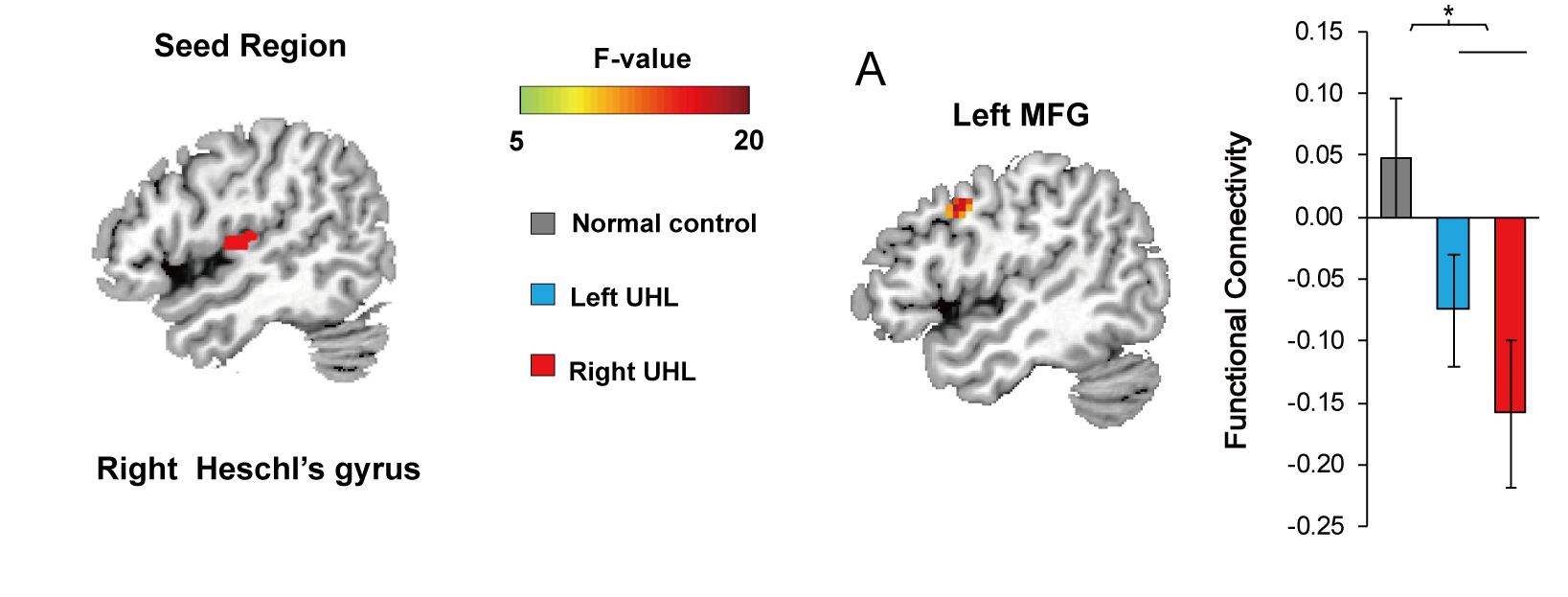

Supplement: Figure S5 — Group difference in resting state functional connectivity with right HG (the seed region) revealed by ANCOVA. The significant differences were shown in left medial frontal gyrus (MFG) (A). The bar and error bar represent the mean value and SD, respectively, of the functional connectivity values in the region. * p<0.05, corrected. (TIF) [file pone.0096126.s005.tif]

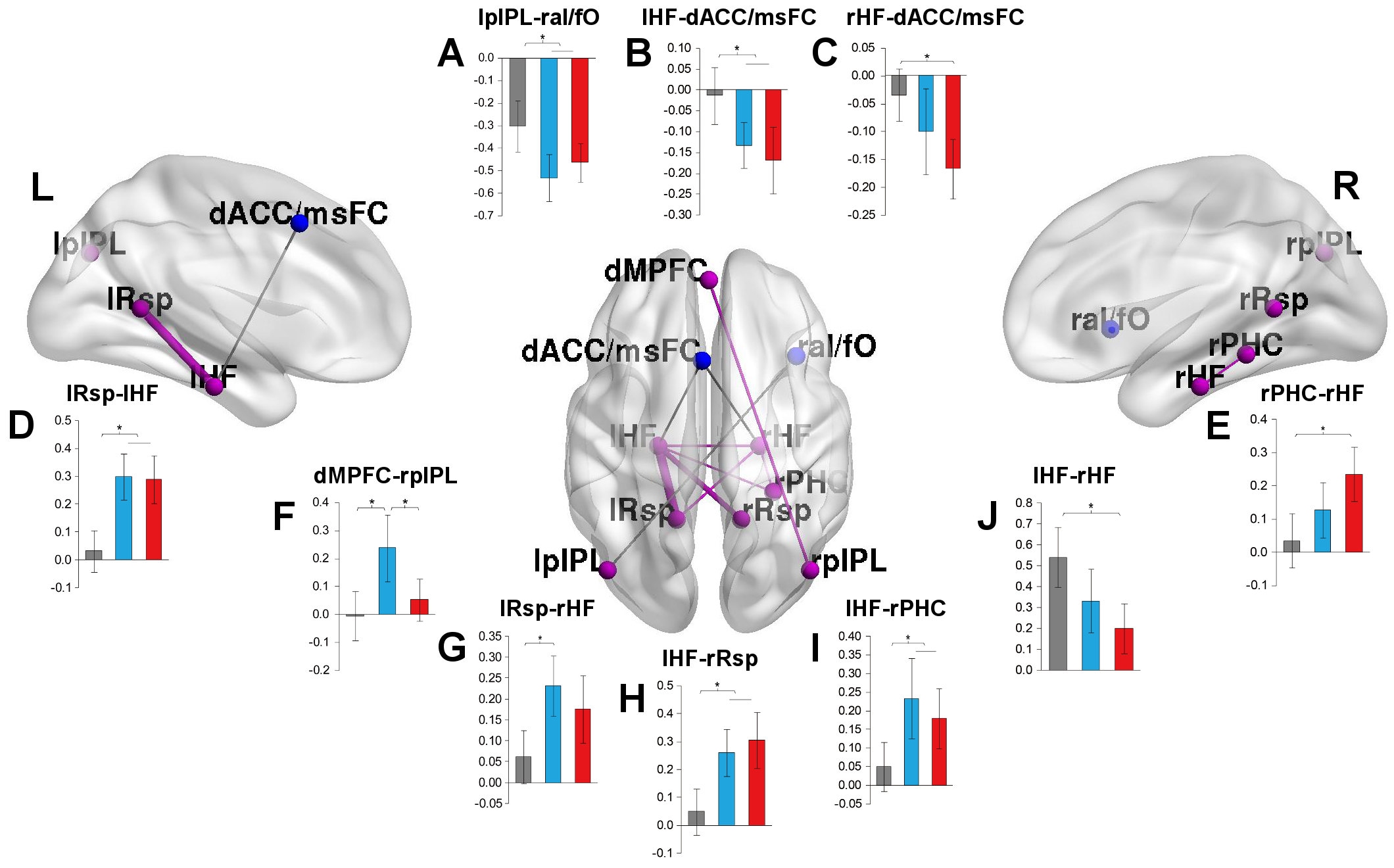

Supplement: Figure S6 — The distribution of connections with significant group effects in the functional connectivity strength among the three groups at p<0.01 (uncorrected). The thicknesss of connections indicate the significance of between-group differences. ROIs in purple belong to DMN while ROIs in blue belong to CCN. Connections in gray indicate those connections were between DMN and CCN (A–C), while connections in purple indicate those connections were within DMN [ipsilateral connetions (D–E), contralateral connections (F–J)]. For each connection, the bar and error bar represent the mean value and SD, respectively, of the functional connectivity strength in each group. Post hoc tests showed that all the ROIs have increased functional connectivity strength in the left UHL patients versus the controls. Three of these six ROI, including the rIPL, rTPJ and rAI/fO showed reduced functional connectivity strength in the right UHL patients compared with the left ones. Only one region (lHF) showed increased functional connectivity strength in right UHL patients compared to normal controls. * p<0.05. The connecitons were mapped onto the cortical surfaces using in-house BrainNet viewer software (www.nitrc.org/projects/bnv/). (TIF) [file pone.0096126.s006.tif]

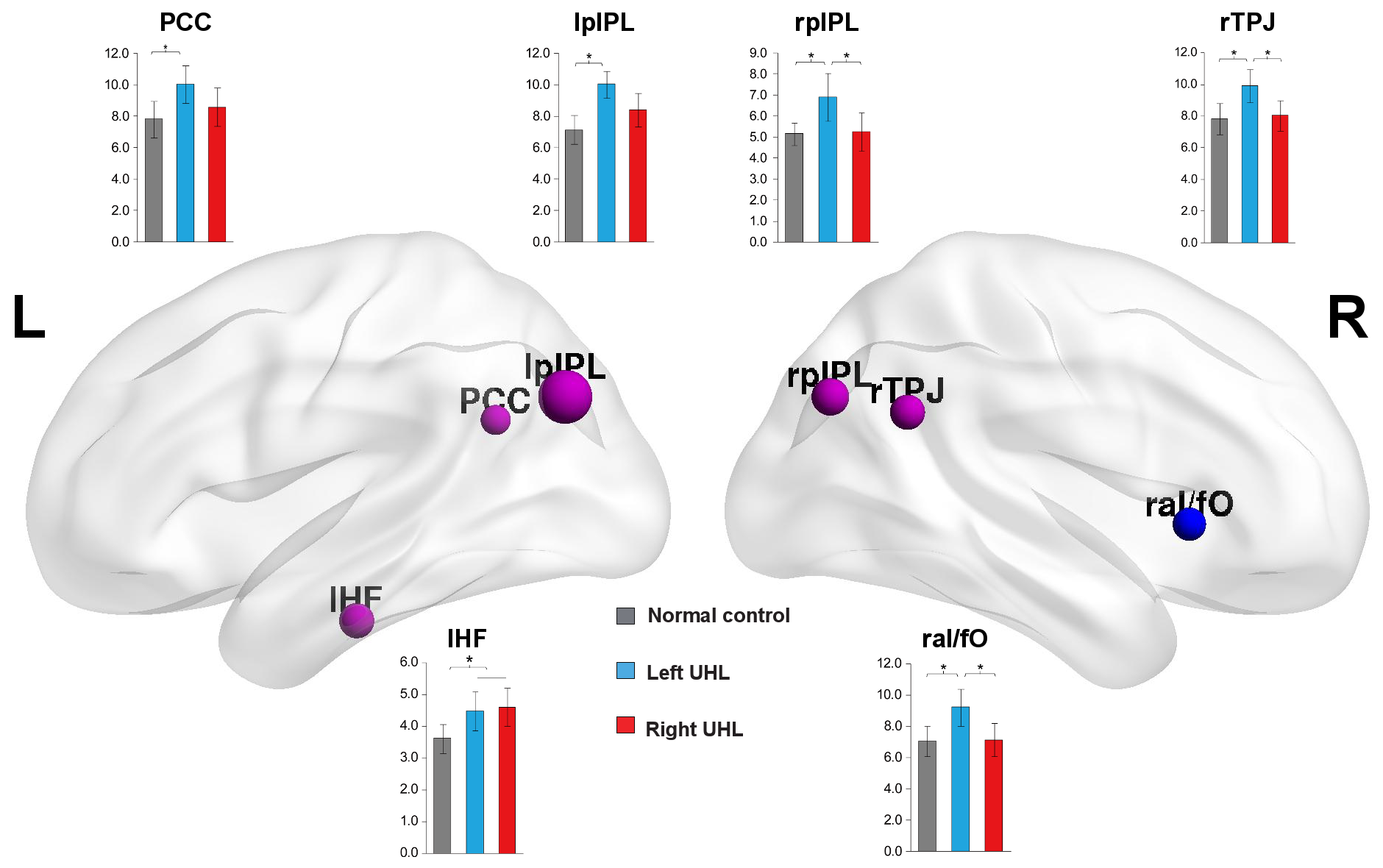

Supplement: Figure S7 — The distribution of brain regions with significant group effects in the functional connectivity strength among the three groups at p<0.05 (uncorrected). The sizes of ROIs indicate the significance of between-group differences. ROIs in purple belong to DMN while ROIs in blue belong to CCN. For each ROI, the bar and error bar represent the mean value and SD, respectively, of the functional connectivity strength in each group. Post hoc tests showed that all the ROIs have increased functional connectivity strength in the left UHL patients versus the controls. Three of these six ROI, including the rIPL, rTPJ and rAI/fO showed reduced functional connectivity strength in the right UHL patients compared with the left ones. Only one region (lHF) showed increased functional connectivity strength in right UHL patients compared to normal controls. * p<0.05. The ROIs were mapped onto the cortical surfaces using in-house BrainNet viewer software (www.nitrc.org/projects/bnv/). For the abbreviations of the ROIs, see Table S1. (TIF) [file pone.0096126.s007.tif]
