# Supplementary material for: Altered Regional and Circuit Resting-State Activity Associated with Unilateral Hearing Loss
Source: PLoS One. 2014 May 1;9(5):e96126. doi: 10.1371/journal.pone.0096126 (PMC4006821; doi:10.1371/journal.pone.0096126)
Supplement: Table S1 — Regions of interest in graph analysis. (DOC) [file pone.0096126.s008.doc]

**Table S1. Regions** of interest in graph analysis

| Region | Abbreviation | L/R | x | y | z |
| --- | --- | --- | --- | --- | --- |
| **Default Mode Network** | | | | | |
| Anterior medial prefrontal cortex | aMPFC | **--** | -6 | 52 | -2 |
| Posterior cingulate cortex | PCC | **--** | -8 | -56 | 26 |
| Dorsal medial prefrontal cortex | dMPFC | **--** | 0 | 52 | 26 |
| Temporal parietal junction | TPJ | L | -54 | -54 | 28 |
|  |  | R | 54 | -54 | 28 |
| Lateral temporal cortex | LTC | L | -60 | -24 | -18 |
|  |  | R | 60 | -24 | -18 |
| Temporal pole | TempP | L | -50 | 14 | -40 |
|  |  | R | 50 | 14 | -40 |
| Ventral medial prefrontal cortex | vMPFC | **--** | 0 | 26 | -18 |
| Posterior inferior parietal lobule | pIPL | L | -44 | -74 | 32 |
|  |  | R | 44 | -74 | 32 |
| Retrosplenial cortex | Rsp | L | -14 | -52 | 8 |
|  |  | R | 14 | -52 | 8 |
| Parahippocampal cortex | PHC | L | -28 | -40 | -12 |
|  |  | R | 28 | -40 | -12 |
| Hippocampal formation | HF | L | -22 | -20 | -26 |
|  |  | R | 22 | -20 | -26 |
| **Cinguloopercular Network** | | | | | |
| Anterior insula/frontal operculum | AI/fO | L | -35 | 14 | 5 |
|  |  | R | 36 | 16 | 4 |
| Dorsal anterior cingulate cortex/ medial | dACC/msFC | **--** | -1 | 10 | 46 |
| superior frontal cortex |  |  |  |  |  |
| Anterior prefrontal cortex | aPFC | L | -28 | 51 | 15 |
|  |  | R | 27 | 50 | 23 |
| Anterior thalamus | aTha | L | -12 | -15 | 7 |
|  |  | R | 10 | -15 | 8 |

Note: Coordinates in this table are based on the Montreal Neurological Institute (MNI) coordinate system. Regions are spheres with 8 mm radius, except that the radius of bilateral anterior thalamus are 6 mm.
