# Supplementary material for: Altered Regional and Circuit Resting-State Activity Associated with Unilateral Hearing Loss
Source: PLoS One. 2014 May 1;9(5):e96126. doi: 10.1371/journal.pone.0096126 (PMC4006821; doi:10.1371/journal.pone.0096126)
Supplement: Table S2 — Group difference in functional connectivity with seed regions at primary auditory cortex. (DOC) [file pone.0096126.s009.doc]

**Table S2. Group difference in functional connectivity with seed regions at primary auditory cortex**

| Seed | Location | Peak | MNI coordinate (mm) | | | Volume |
| --- | --- | --- | --- | --- | --- | --- |
|  |  | F-value | x y z | | | (mm^3^) |
| Left HG | left MFG | 13.46 | -45 | 9 | 39 | 1409 |
|  | right SPL | 15.47 | 27 | -57 | 69 | 459 |
| Right HG | left MFG | 12.22 | -51 | 6 | 42 | 648 |

Abbreviations: HG = Heschl’s gyrus , MPG = medial frontal gyrus , SPL= superior parietal lobule, UHL = unilateral hearing loss, NC = normal controls.
