# Supplementary material for: Altered Regional and Circuit Resting-State Activity Associated with Unilateral Hearing Loss
Source: PLoS One. 2014 May 1;9(5):e96126. doi: 10.1371/journal.pone.0096126 (PMC4006821; doi:10.1371/journal.pone.0096126)
Supplement: Text S1 — (DOC) [file pone.0096126.s010.doc]

**ROI-based resting-state functional connectivity analysis**

To examine more directly the potential reorganization of brain activity in the DMN and CCN networks subserving higher-order cognitive functions, we performed ROI-based functional connectivity analysis using previously deﬁned ROIs (Table S1) of the DMN and cinguloopercular network, a representative networks that compose the CCN involving rAI . Andrews-Hanna and colleagues defined seven left and four midline ROIs for DMN, and here we additionally defined seven right-lateralized ROIs, mirroring the coordinates of the left ones. We extracted the time series (preprocessed with nuisance signal removal and spatial smoothing in the previous section) for each of the ROIs described above and computed the cross correlation coefficient matrix among the ROIs for each participant. We then converted the correlations to *z* values using Fisher’ *z*-transformation. For the *z*-value between each pair of ROIs, ANCOVA was conducted among the three groups, with age and gender as covariates. For each ROI, functional connectivity strength at this given ROI was computed as the average of absolute functional connectivity between this ROI and all other ROIs. Similar ANCOVA was conducted on functional connectivity strength of each ROI among the three groups.

As shown in Fig. S6, ten out of three hundred connections showed group difference (*p* < 0.01 uncorrected), with the strength of resting-state functional connectivity increased between DMN and CCN (Figs. S6A – S6C) and within DMN (Figs. S6D – S6J) in UHL patients (except for the connection between bilateral HF, for which right UHL showed decreased connectivity). Importantly, the increased strength of resting-state functional connectivity within DMN between the left retrosplenial cortex and left hippocampal formation in UHL patients were significant after Bonferroni correction (p = 8.5*10^-6^ < 0.05/300). No connection within the CCN was found to be altered in UHL. Five out of eighteen DMN ROIs and one out of seven CCN ROIs exhibited group difference (*p* < 0.05, uncorrected) amongst the three groups (Fig. S7), with the UHL patients showing increased connectivity strength in all six ROIs. Importantly, the increased strength of connectivity in the DMN, i.e. the left posterior inferior parietal lobule in UHL patients were significant after Bonferroni correction (p = 6.6*10^-4^ < 0.05/25).

**Resting-state functional connectivity (RSFC) of the primary auditory cortex**

Importantly, as the auditory cortex is critical for auditory perception and may reflect the functional state of the hearing directly. We also conducted similar RSFC analysis seeding at the bilateral primary auditory cortex (Heschl’s gyrus) respectively. Multiple comparison correction was performed according to the Gaussian Random Field theory with a corrected p < 0.05 (uncorrected p < 0.001 and minimum 21 voxels in a cluster) within the whole brain mask.

The left medial frontal gyrus (MFG) and right superior parietal lobule (SPL) showed significant group difference among the three groups of participants on the connectivity strength of the left GH (Figure S4). The negative RSFC between SPL and left HG were decreased in both left and right UHL participant compared with NC, and the negative connectivity between left HG and MFG were increased. Meanwhile, the significant different RSFC were also shown between right HG and left MFG (Figure S5), and the positive connectivity were turned out to negative.

Previous studies have shown that deaf people have better visual performance and enhanced peripheral visual attention across time or space. Accordingly, such alteration involved with attentional cognitive control areas may suggest that the attenuation of hearing information input leads to a functional in-coordination among auditory perception and goal-directed attention.

References:

1. Andrews-Hanna JR, Reidler JS, Sepulcre J, Poulin R, Buckner RL (2010) Functional-anatomic fractionation of the brain's default network. Neuron 65: 550-562.

2. Fan J, Van Dam, N. T., Gu, X., Liu, X., Wang, H., Cheuk, Y. T., Hof, P (2013) Quantitative Characterization of Functional Anatomical Contributions to Cognitive Control under Uncertainty. Journal of Cognitive Neuroscience,.

3. Dosenbach NU, Fair DA, Miezin FM, Cohen AL, Wenger KK, et al. (2007) Distinct brain networks for adaptive and stable task control in humans. Proc Natl Acad Sci U S A 104: 11073-11078.

4. Bavelier D, Dye MW, Hauser PC (2006) Do deaf individuals see better? Trends Cogn Sci 10: 512-518.

5. Dye MW, Baril DE, Bavelier D (2007) Which aspects of visual attention are changed by deafness? The case of the Attentional Network Test. Neuropsychologia 45: 1801-1811.

6. Proksch J, Bavelier D (2002) Changes in the spatial distribution of visual attention after early deafness. J Cogn Neurosci 14: 687-701.

7. Weisberg J, Koo DS, Crain KL, Eden GF (2012) Cortical plasticity for visuospatial processing and object recognition in deaf and hearing signers. Neuroimage 60: 661-672.

8. Daza MT, Phillips-Silver J (2013) Development of attention networks in deaf children: Support for the integrative hypothesis. Res Dev Disabil 34: 2661-2668.
